# Magnetic Resonance Angiography Using Fresh Blood Imaging in Oral and Maxillofacial Regions

**DOI:** 10.1155/2012/865369

**Published:** 2012-10-17

**Authors:** Masafumi Oda, Tatsurou Tanaka, Shinji Kito, Manabu Habu, Masaaki Kodama, Shinya Kokuryo, Ikuya Miyamoto, Daigo Yoshiga, Kensuke Yamauchi, Shinnosuke Nogami, Nao Wakasugi-Sato, Shinobu Matsumoto-Takeda, Ayataka Ishikawa, Ikuko Nishida, Katsura Saeki, Kazumasa Morikawa, Kou Matsuo, Yuji Seta, Yoshihiro Yamashita, Kenshi Maki, Kazuhiro Tominaga, Yasuhiro Morimoto

**Affiliations:** ^1^Department of Oral Diagnostic Science, Kyushu Dental College, 2-6-1 Manazuru, Kokurakita-ku, Kitakyushu 803-8580, Japan; ^2^Department of Oral and Maxillofacial Surgery, Kyushu Dental College, 2-6-1 Manazuru, Kokurakita-ku, Kitakyushu 803-8580, Japan; ^3^Department of Pathology, Saitama Cancer Center, Saitama 362-0806, Japan; ^4^Department of Growth and Development of Function, Kyushu Dental College, 2-6-1 Manazuru, Kokurakita-ku, Kitakyushu 803-8580, Japan; ^5^Department of Oral Bioscience, Kyushu Dental College, 2-6-1 Manazuru, Kokurakita-ku, Kitakyushu 803-8580, Japan; ^6^Department of Oral and Maxillofacial Surgery, Fukuoka Dental College, Fukuoka 814-0193, Japan; ^7^Center for Oral Biological Research, Kyushu Dental College, 2-6-1 Manazuru, Kokurakita-ku, Kitakyushu 803-8580, Japan

## Abstract

The present paper provides general dentists with an introduction to the clinical applications and significance of magnetic resonance angiography (MRA) in the oral and maxillofacial regions. Specifically, the method and characteristics of MRA are first explained using the relevant MR sequences. Next, clinical applications to the oral and maxillofacial regions, such as identification of hemangiomas and surrounding vessels by MRA, are discussed. Moreover, the clinical significance of MRA for other regions is presented to elucidate future clinical applications of MRA in the oral and maxillofacial regions.

## 1. Introduction

It is very important and significant for doctors to determine the status of vascular-related diseases such as thrombosis and arteriosclerosis and to precisely visualize the three-dimensional (3D) structure of the vasculature before surgery [1–3]. In dental fields, understanding the 3D structure of the vasculature is necessary and important for surgical operations on tumors and cysts. The most precise evaluation technique is conventional angiography (CR) using contrast medium. In CR, if the treatment should be applied for the status of vessels, the technique was continuously followed by the interventional radiology such as the embolization of arteries with arteriosclerosis. Therefore, the CR is a firm position for elucidating the status of vascular-related disease such as thrombosis and arteriosclerosis, and precisely visualization of 3D vasculatures at the preoperations. However, the risk of a new focal neurological deficit caused by CR is about 0.14% ~ 0.5%, and this risk rises to 1.3% ~ 2.6% in patients investigated for stroke and transient ischemic attacks [4]. Therefore, it is important to identify the 3D structure of the vasculature of the arterial and venous systems as noninvasively as possible. To determine the 3D structure of the vasculature as noninvasively as possible, ultrasonography (US) is easy to use clinically. However, US cannot objectively visualize a wide area. Furthermore, the 3D structure of the vasculature cannot be visualized using computed tomography (CT) without contrast medium. Of course, CT angiography (CTA) using contrast medium was a very useful tool for the 3D structure of the vasculature. However, the risk of complications in CTA should be higher because of iodine contrast medium.

Recent advances in magnetic resonance imaging (MRI), 3D data acquisition, and postprocessing technologies are expanding the potential applications of 3D displays [5–16]. As a noninvasive technique, magnetic resonance angiography (MRA) has been developed to identify and characterize peripheral blood vessels located in the thoracic and abdominal regions and the lower extremity [17–22]. Of course, MRA is very useful to visualize the 3D structure of the vasculature in the oral and maxillofacial regions, and it is clinically significant for evaluating the relationship between lesions and vessels in the oral region [14, 16, 23–26]. General dentists should know and easily understand the clinical applications of MRA in the oral and maxillofacial regions.

Therefore, the present paper provides a clear and easily understandable explanation of MRA, the method, and its characteristics, as well as its clinical applications and characteristics, for general dentists.

## 2. Explanation of MRA, Its Method, and Characteristics Using the Relevant MR Sequences

MRA commonly uses a bright-blood method, in which the signal from the moving protons is accentuated relative to the stationary protons through pulse sequences and measurement parameters. In fact, MRA is a technique using vessel flow or contrast agents such as gadolinium to detect vessel systems such as arteries and veins. Therefore, MRA has the least complication and is the easiest technique for 3D visualization of vessels, unlike CR using contrast medium such as iodine-related medium. At the same time, this technique is very useful for identifying vascular diseases and abnormalities, as well as the relationships between diseases and surrounding vessels.

The various techniques of MRA include MR sequences such as time of flight (TOF), phase contrast (PC), fresh blood imaging (FBI), and contrast-enhanced MRA [5–11, 13, 15, 16, 27–37]. MRA using TOF (TOF-MRA) is the most time-efficient method for obtaining MRA images. A single measurement is performed, with the stationary tissue signal suppressed relative to the flowing tissue signal [38]. MRA using PC (PC-MRA) is a technique in which the background tissue signal is subtracted from the flow-enhanced image to produce flow-only images, analogous to digital subtraction X-ray angiography [38]. MRA as a new technique using FBI that has been applied to the 3D evaluation of vasculature [8–11, 13, 15, 16, 34–37]. FBI, which uses 3D half-Fourier fast advanced spin echo (FASE)-triggered MR with electrocardiographic (ECG) gating, is one of the newest non-enhanced-MRA techniques [8–11, 13, 15, 16, 34–37]. Contrast-enhanced MRA makes good use of the contrast agent, which affects the relaxation times of the water protons in the nearby tissue. In MRA with contrast medium, as disadvantages, intravenous injection should be applied such as CR, and gadolinium could induce many kinds of complications including nephrogenic fibrosing dermopathy. MRA without contrast medium has no complications and no need of intravenous injections, and its technique can easily and additionally apply for many patients in whom vascular information should be evaluated. In the present paper, the focus is on three techniques (TOF, PC, and particularly FBI) as the main MRA techniques without contrast medium that are useful in the oral and maxillofacial regions.

## 3. Clinical Applications of MRA for Lesions, Mainly Hemangiomas, in the Oral and Maxillofacial Regions

**Figure 1:**
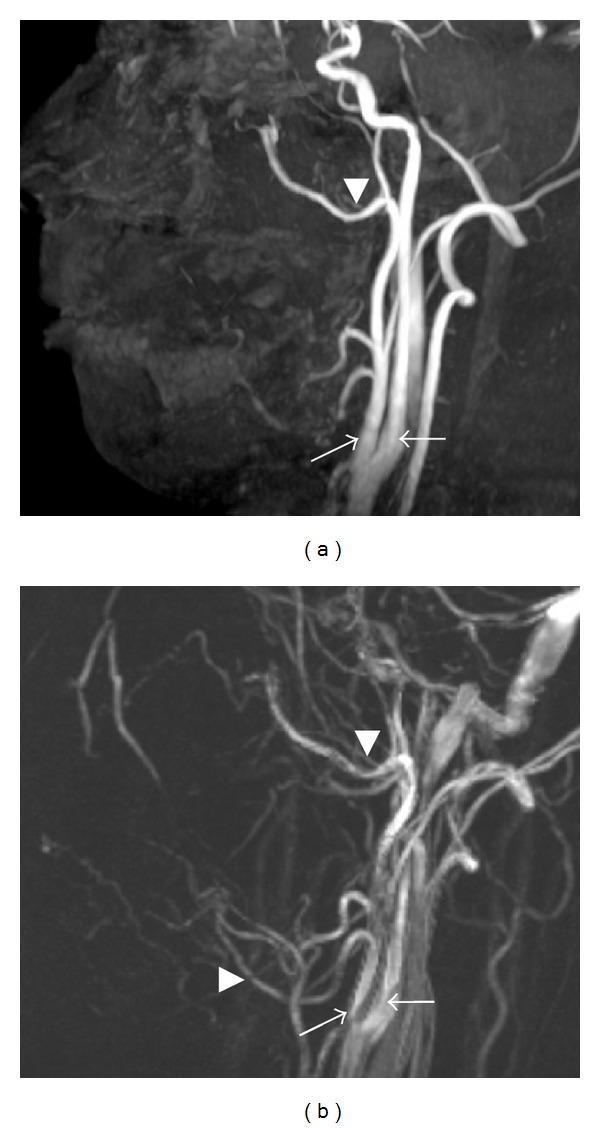
MRA using 3D-TOF (a) and 3D-PC (b) in the oral and maxillofacial regions of a healthy 33-year-old volunteer. (a) On MRA using 3D-TOF, mainly the carotid, maxillary, and facial arteries are shown clearly, but not its branches. The external carotid artery (arrow), internal carotid artery (black arrow), maxillary artery (arrowhead), and facial artery (black arrowhead). Acquisition time was 6 minutes and 39 seconds. (b) On MRA using 3D-PC, the carotid, maxillary, facial, and other peripheral arteries are shown in the same subject as in Figure 1(a). The external carotid artery (arrow), internal carotid artery (black arrow), maxillary artery (arrowhead), and facial artery (black arrowhead). Acquisition time was 10 minutes and 15 seconds.

On TOF- or PC-MRA, arteries and veins which are to some degree slow appear as bright homogeneous linear structures and stationary tissues except vessels with flow which appear for the most part blurred(Figure 1). Maximum intensity projection and multiplanar reconstruction techniques are used to visualize MRA data. Therefore, vessels with flow can be primarily visualized on MRA with these two techniques (Figure 1). On the other hand, vessels without flow cannot be visualized at all using MRA with TOF and PC techniques.

**Figure 2:**
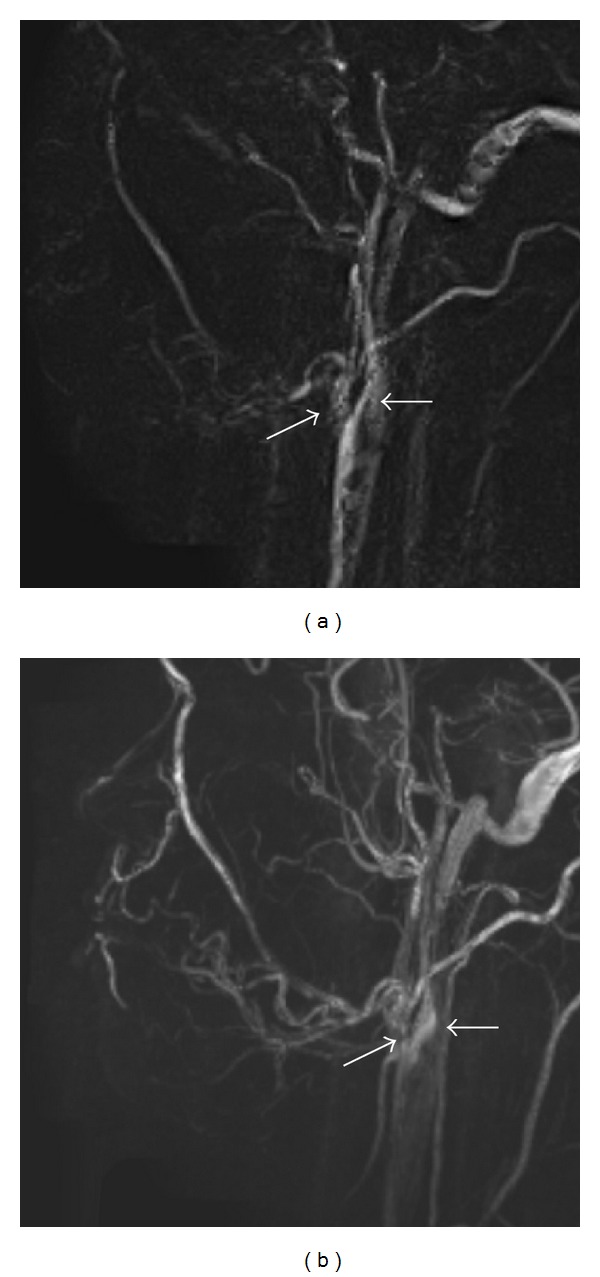
MRA using 2D-PC (a) and 3D-PC (b) in the oral and maxillofacial regions of a healthy 28-year-old volunteer. Three-dimensional (3D) PC-MRA has a better signal-to-noise ratio (SNR) and a better spatial resolution than 2D PC-MRA. (a) On MRA using 2D-PC, mainly the external (arrow) and internal carotid arteries (black arrow) and their branches are shown, but the image quality is low. Acquisition time was 9 minutes and 36 seconds. (b) On MRA using 3D-PC, the external (arrow) and internal carotid (black arrow) and many other branches are seen. The images are higher in quality in the same subject as in Figure 2(a). Acquisition time was 10 minutes and 15 seconds.

Of the two techniques in use for patients, PC-MRA is better for demonstrating slow-flowing blood vessels (<10 cm/s), owing primarily to an optimized velocity-encoding gradient (VENC) and perhaps also to smaller saturation effects and better background suppression [39]. This sequence offers a better examination of the external carotid system than TOF-MRA [39]. Though the main carotid artery and its branches can be visualized on PC-MRA, TOF-MRA can visualize the main carotid artery, but not its branches (Figure 1) [14]. In addition, 3D-PC-MRA has a better signal-to-noise ratio and a better spatial resolution than 2D-PC-MRA (Figure 2) [40]. Therefore, in the oral and maxillofacial regions, 3D-PC-MRA routinely tends to be selected for additional and preoperative vascular evaluations in MR examinations.

However, in the oral and maxillofacial regions, the vasculature is complex, with blood flow rates varying from high in the carotid arteries to low in the lingual arteries. Thus, 3D visualization of the vasculature in these regions is very difficult to obtain even using 3D-PC-MRA. Therefore, in very recent reports, MRA using FBI (FBI-MRA) was used for the 3D evaluation of the vasculature in the oral and maxillofacial regions [10, 16, 23]. Most prior studies using FBI primarily examined the main vessels of the chest, abdomen, and lower extremities [9, 11, 13, 15, 34–37].

**Figure 3:**
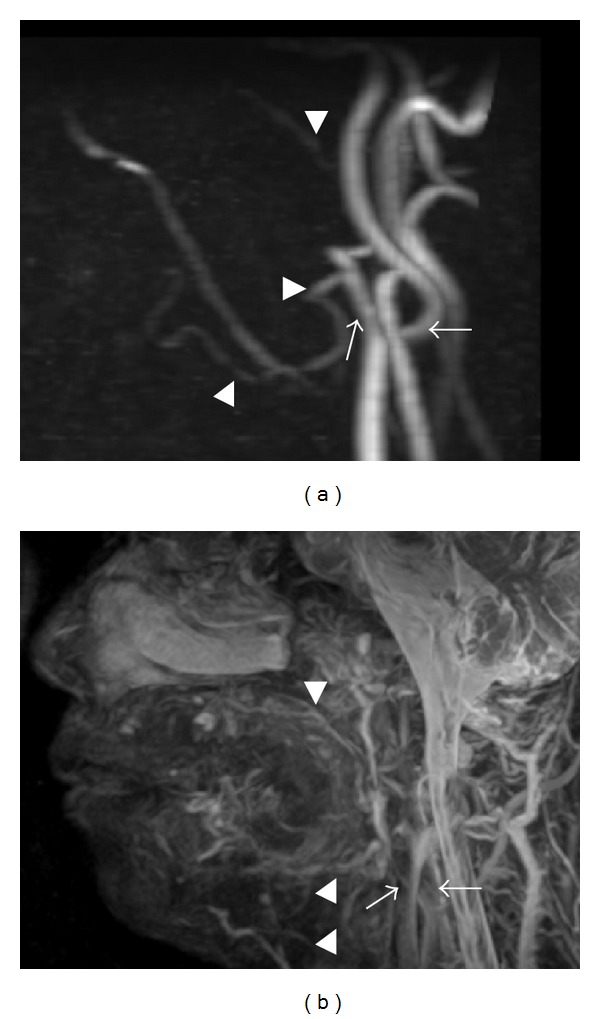
MRA using 3D-PC (a) and 3D-FBI (b) in the oral and maxillofacial regions of a healthy 48-year-old volunteer. MRA using 3D-FBI provides better 3D visualization of the vasculature of thin blood vessels, including the lingual and facial arteries, than 3D-PC-MRA. (a) On MRA using 3D-PC, the external (arrow) and internal carotid (black arrow), maxillary (arrowhead), and facial arteries (black arrowhead) are shown. Acquisition time was 10 minutes and 15 seconds. (b) On MRA using 3D-FBI, the external (arrow) and internal carotid (black arrow) and other peripheral vessels are shown. The maxillary (arrowhead), facial (black arrowhead), lingual (gray arrowhead), and further peripheral arteries are more precisely shown than in Figure 3(a) in the same subject. Acquisition time was 4 minutes and 5 seconds.

In previous reports, 3D-FBI-MRA could provide better 3D visualization of the vasculature of thin blood vessels, including the lingual and facial arteries, than 3D-PC-MRA (Figure 3) [16]. The axes of the lingual and facial arteries are under 2-3 mm. 3D-FBI-MRA permits the depiction of slow-flowing blood vessels by acquiring the images with ECG triggering during the slow-flow cardiac phase [31]. 3D-FBI-MRA can depict slow-flowing vessels due to the T2 effects, so that even relatively static blood vessels can be depicted on images, unlike imaging with 3D-PC-MRA [31]. Therefore, 3D-FBI-MRA can even visualize vessels with very slow flow that cannot be visualized on 3D-PC-MRA [7, 9, 13, 16]. In addition, the depiction level of thick blood vessels such as external and internal carotid arteries using 3D-FBI is the same as that using 3D-PC-MRA. In other word, peripheral small arteries look well visualized on 3D-FBI-MRA compared to 3D-PC-MRA, but not for large arteries. Carotid, maxillary, facial, and lingual arteries can be more precisely visualized on 3D-FBI-MRA without contrast medium than on 3D-PC-MRA (Figure 3). At the same time, the internal jugular, maxillary, facial, and lingual veins can also be better visualized on 3D-FBI-MRA without contrast medium than on 3D-PC-MRA (Figure 3).

**Figure 4:**
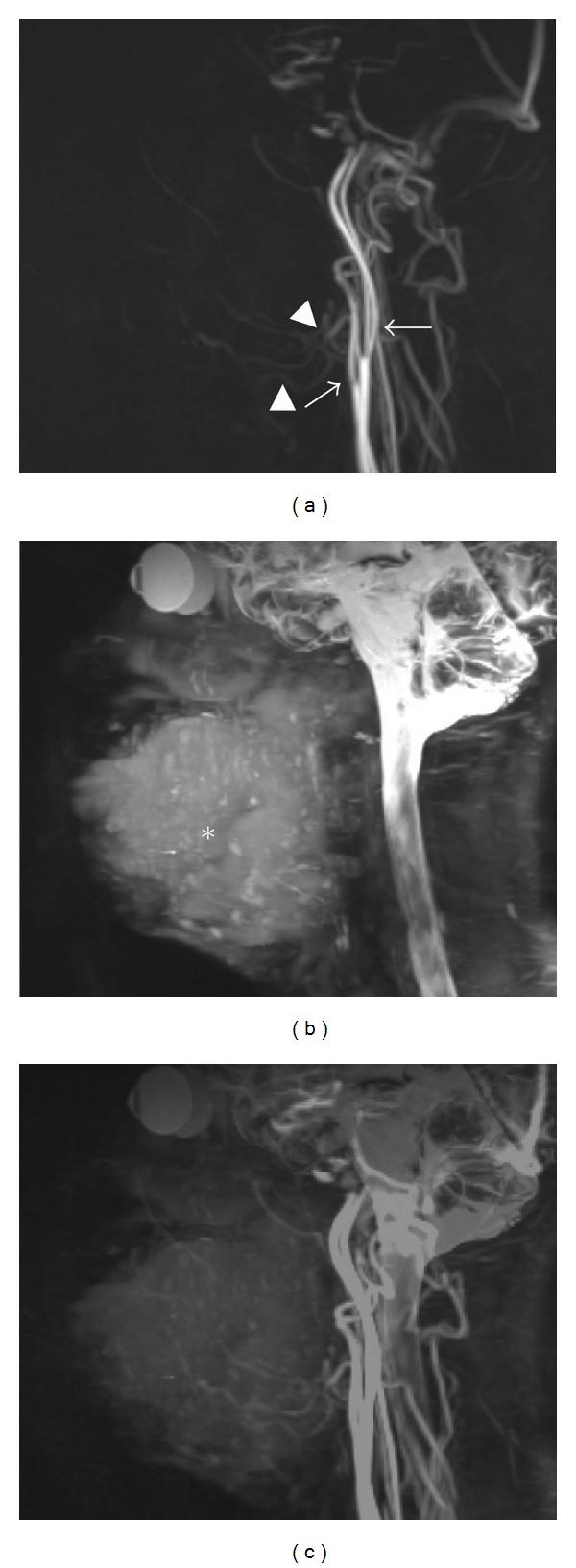
The production of a superimposed view of MRA using 3D-PC and a T2-weighted image in a 46-year-old patient with multiple hemangiomas of the bilateral tongue, lower lip, cheek, oral floor, masticator space, and right parotid space. (a) On MRA using 3D-PC, the external (arrow) and internal carotid arteries (black arrow), and the facial (arrowhead) and lingual arteries (black arrowheads) are shown, but the hemangiomas in the orofacial area are not seen. Acquisition time was 10 minutes and 15 seconds. (b) Fat-saturation T2-weighted image using 3D-FASE sequences of the head and neck. A hemangioma is visualized on this image (star). Acquisition time was 5 minutes and 8 seconds. (c) On fusing fat-saturation T2-weighted images with MRA by 3D-PC, hemangiomas and surrounding vessels can be visualized. However, image resolution of the fusion image is degraded compared to that of MRA using 3D-PC.

3D-TOF- or PC-MRA depicts only blood vessels, but not mass lesions such as hemangiomas (Figure 4) [16, 23]. Therefore, the 3D relationship between the mass lesions such as hemangiomas and surrounding blood vessels should be visualized by fusing fat-saturation T2-weighted images with 3D-PC-MRA [14]. By doing so, hemangiomas and surrounding vessels can be visualized on the fusion images (Figure 4). The software to allow superimposition of both 3D-PC-MRA and T2-weighted images is integral to the MR system. However, image resolution degradation is a disadvantage of fusion imaging (Figure 4). Deterioration of the contrast-to-noise and signal-to-noise ratios on superimposed views occurs because noise on the respective images is doubled without increasing the signal intensity of the tumor and vessels [14]. In addition, production of the superimposed view might be unsuccessful if the patient should move in the time gap between capture of the respective images [14].

**Figure 5:**
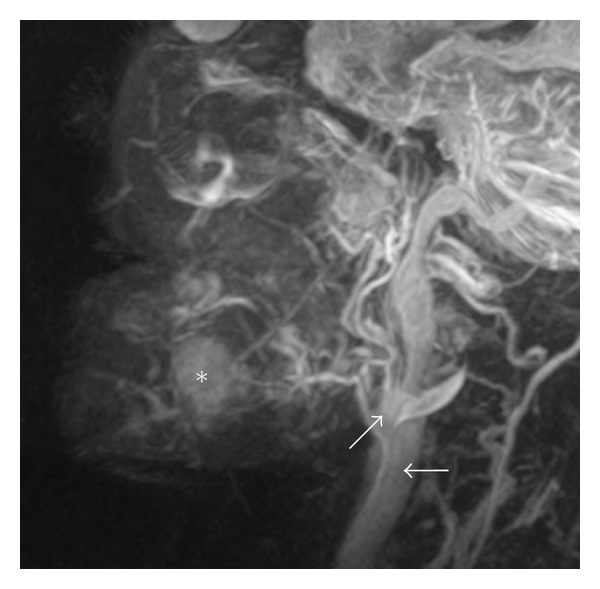
MRA by 3D-FBI in a 79-year-old patient with hemangiomas of the left oral floor. Both hemangiomas and thin blood vessels around tumor can be clearly visualized on MRA with 3D-FBI. The carotid arteries (arrow), internal jugular vein (black arrow), lingual artery, lingual vein, facial artery, facial vein, maxillary artery, and maxillary vein are identified as bright homogeneous linear structures. At the same time, hemangiomas (star) are clearly visualized as the mass with a high signal intensity. Acquisition time was 3 minutes and 28 seconds.

On the other hand, both hemangiomas and thin blood vessels around tumors can be clearly visualized on 3D-FBI-MRA without fusion of MRA with tumor images (Figure 5) [23]. The carotid arteries, internal jugular veins, lingual arteries, lingual veins, facial arteries, facial veins, maxillary arteries, and maxillary veins can be seen as bright homogeneous linear structures (Figure 5). At the same time, hemangiomas are clearly visualized as masses with high signal intensity (Figure 5). Except for the carotid arteries, 3D-FBI-MRA is better than 3D-PC-MRA based on visual scores despite the short acquisition time [23]. Artifacts from patient movement and shortened acquisition times reduce image quality more with 3D-PC-MRA than with 3D-FBI.

**Figure 6:**
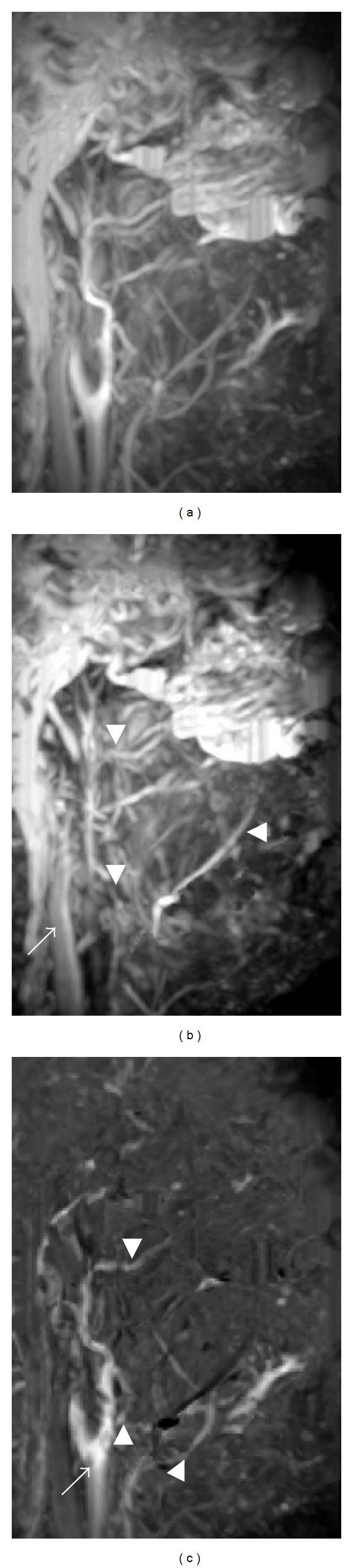
MRA by 3D-FBI in the oral and maxillofacial regions of a 49-year-old healthy volunteer. MRA using 3D-FBI can distinguish between arteries (a) and veins (b) without the use of contrast medium. (a) Images acquired during diastole. The carotid artery, internal jugular vein, maxillary artery, maxillary vein, facial artery, facial vein, lingual artery, and lingual vein are visualized. Acquisition time was 3 minutes and 36 seconds. (b) Images acquired during systole. The internal jugular vein (arrow), maxillary vein (arrowhead), facial vein (black arrowhead), and lingual vein (gray arrowhead) are depicted. Acquisition time was 3 minutes and 36 seconds. (c) The subtraction image of the systolic image (b) from the diastolic image (a) that depicts the carotid (arrow), maxillary (arrowhead), facial (black arrowhead), and lingual arteries (gray arrowhead).

Another advantage of 3D-FBI-MRA is that one can distinguish between arteries and veins without the use of contrast medium [8–11, 13, 15, 16, 23, 34–37]. The blood vessels depicted on 3D-FBI-MRA are most of the arteries, including the carotid, maxillary, facial, lingual, and other arteries (Figure 6). Flow-spoiled gradient pulses do not affect the signal intensity of stationary background tissues. Veins are similarly less affected by the flow-spoiled pulse during diastole and systole as a result of their relatively constant slow flow throughout the cardiac cycle [15]. By applying the flow-spoiled pulses, the signal intensity difference between diastole and systole in the arteries is increased. Thus, diastolic and systolic subtraction provides better delineation of the arteries. However, except for 3D-FBI, the distinction between arteries and veins has not been possible with MRA sequences such as 3D-TOF and 3D-PC without contrast medium so far. It is hoped that 3D-FBI-MRA will be a significant, epoch-making technique.

However, there are some potential disadvantages with 3D-FBI-MRA for clinical applications in the oral and maxillofacial regions [16, 34–37]. The first is that the selection of the appropriate flow-spoiled gradient is important for determining the image quality of hemangiomas and surrounding vessels. Even a slight mistake affects the visualization of branch arteries, such as the maxillary and lingual arteries [16, 23]. To select the appropriate flow-spoiled gradient pulses is very difficult for operators, because blood vessels in the oral and maxillofacial regions have a wide range of flow rates, from high to static. The second disadvantage is that 3D-FBI-MRA is sensitive to movement. Therefore, the images tend to be degraded in patients with movements of the mandible and tongue. Moreover, the third disadvantage is that patients with arrhythmias or tachycardias are not suitable candidates for FBI examinations, because ECG triggering is used.

## 4. Future Uses of MRA for Lesions in the Oral and Maxillofacial Regions

MRA cannot perfectly replace CR for examinations of vascular-related diseases and suspected hemangiomas in the oral and maxillofacial regions. That is why MRA cannot have more contrast between vessels and tissues except vessels and cannot be more precisely visualized than CR. However, performing MRA for diseases in the oral and maxillofacial regions should be recommended in addition to common MR examinations before CR. In particular, 3D-FBI-MRA is relatively easy to perform and can precisely visualize thin vessels except a little difficulty of parameters setting. Its technique can distinguish between arteries and veins without the use of contrast medium. Therefore, examinations using 3D-FBI-MRA are very useful and important for preoperative evaluations for various kinds of diseases, in particular the 3D relationship between the vasculature and diseases. MRA with contrast medium is also important. At present, higher spatial resolution gadolinium-MRA can be achieved at 3 Tesla with a sustained or greater signal-to-noise ratio of enhanced vasculature, relative to 1.5 Tesla [41]. Therefore, MRA with higher resolution in the oral and maxillofacial regions can be achieved with a 3-Tesla MR system. Very recently, it has been found that existing MRA is suboptimal for assessing the hemodynamics of arteriovenous malformations [42–50]. As a completely noninvasive method, 4D dynamic MRA offers hemodynamic information with a temporal resolution of 50–100 ms for the evaluation of arteriovenous malformations and can complement existing methods, such as dynamic subtraction analysis and TOF MRA [42–46]. Therefore, the evaluation of hemodynamics in hemangiomas will be performed by MRA in the oral and maxillofacial regions in the future.
